# Advances in Fabrication Materials of Honeycomb Structure Films by the Breath-Figure Method

**DOI:** 10.3390/ma6020460

**Published:** 2013-02-04

**Authors:** Liping Heng, Bin Wang, Muchen Li, Yuqi Zhang, Lei Jiang

**Affiliations:** 1 Key Laboratory of Organic Solids, Center for Molecular Science, Institute of Chemistry, Chinese Academy of Sciences, Beijing 100190, China; E-Mails: limc0104@163.com (M.L.); jianglei@iccas.ac.cn (L.J.); 2 School of Environment, Tsinghua University, Beijing 100084, China; E-Mail: thuwb@tsinghua.edu.cn; 3 College of Chemistry and Chemical Engineering, Yan’an University, Yan’an, Shaanxi 716000, China;

**Keywords:** honeycomb structure, fabrication material, breath-figure method, pattern structure, hexagonal geometry structure, polymer film, hybrid film, small organic molecule, nanoparticle

## Abstract

Creatures in nature possess almost perfect structures and properties, and exhibit harmonization and unification between structure and function. Biomimetics, mimicking nature for engineering solutions, provides a model for the development of functional surfaces with special properties. Recently, honeycomb structure materials have attracted wide attention for both fundamental research and practical applications and have become an increasingly hot research topic. Though progress in the field of breath-figure formation has been reviewed, the advance in the fabrication materials of bio-inspired honeycomb structure films has not been discussed. Here we review the recent progress of honeycomb structure fabrication materials which were prepared by the breath-figure method. The application of breath figures for the generation of all kinds of honeycomb is discussed.

## 1. Introduction

**Figure 1:**
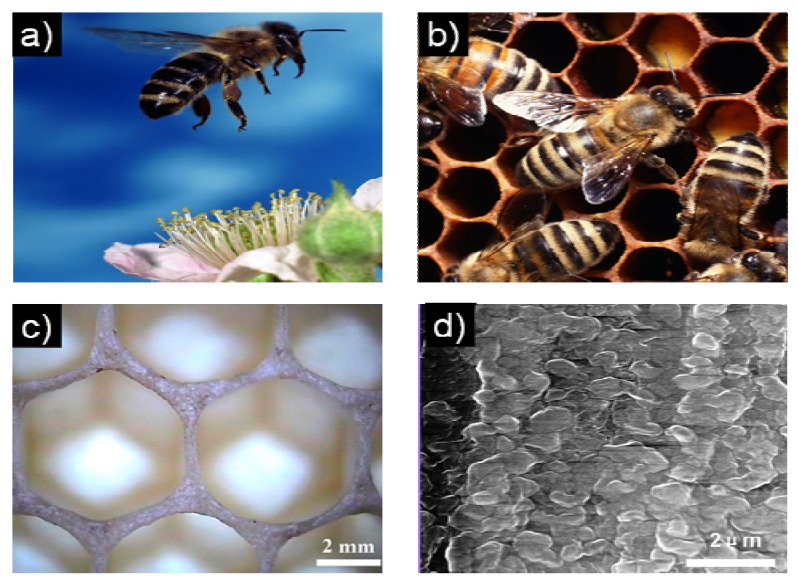
Photos of the **(a)** honeybee; and **(b)** the combs; **(c)** top view of fresh honeycomb walls and cells; **(d)** environmental scanning electron microscope (ESEM) image of a cross section of the cell wall showing wax grains [10].

Honeybees use the minimum beeswax to build natural honeycomb which has a delicate and perfect hexagonal geometrical structure (Figure 1a–b). This honeycomb structure has wonderful properties, such as large space area, good structural stability, high mechanical strength, low density, buffering humidity fluctuations, together with thermal and acoustic insulation [1,2,3,4]. Thus, the honeybee comb is one of the natural cellular structures which has been studied most and has long fascinated mathematicians, physicists, and biologists [5,6,7,8,9,10,11,12,13,14,15]. It is well known that the bees build the combs out of hexagonal cells (Figure 1c) [10]. The detailed microstructures of the walls, wax, silk, and the macroscopic properties of the honeybee combs have been clearly revealed (Figure 1d). Their hexagonal structures for biomimetic designs have been fully explored. The microstructures of biomaterials are increasingly providing a rich route for synthesis of artificial materials with superior properties [16,17,18,19]. In particular, the commonly hierarchical structures in nature can lead to breakthroughs in the design of new materials [20,21]. Natural honeybee combs have long been a paradigm for engineering cellular structures [8].

There are many successful methods for constructing ordered honeycomb structures with controllable pores over a large area. However, these top-down approaches usually involve multiple, complicated, and expensive steps, and may damage the final pattern after removal of the templates. In 1994, Francois *et al*. [22] first reported that using condensed water droplets as a dynamic template, the so-called breath-figure (BF) method, [23,24] can be used to prepare highly ordered honeycomb-patterned films with two- or three-dimensional hexagonal arrays of pores. Compared with other methods, the BF method is a simple one-step and template-free strategy for the organization of ordered honeycomb porous structures. In this method, a solution of polymer is first cast on a solid surface under high humid conditions, and then rapid evaporation of the volatile solvent causes a quick drop in the surface temperature under the dew point, which leads to fast condensation and nucleation of water droplets on the surface of the polymer solution [25]. During evaporation, the water droplets grow slowly under the protection of the polar polymer [26], and pack into a hexagonal pattern due to the convectional flow or the capillary force generated at the solution front [27,28,29]. After complete evaporation of solvent and water, traces of water droplets remain in the polymer film to form honeycomb structures. In the film fabrication process by the BF method, high relative humidity and a volatile solvent are two key factors for the formation of a honeycomb pattern. Other influencing factors [30,31,32] such as molecular weight of polymer, air velocity, concentration, selective solvent, evaporation time, and substrates, have also been used to control the morphologies and properties of the honeycomb films. 

Since the introduction of the BF fabrication method by Francois *et al*. [33], honeycomb films constructed by the BF method have been paid a lot of attention. Several scientists, such as Shimomura and Stenzel, have performed systematic work. They extended the BF method to all kinds of building units, such as starlike polymers [34], block copolymers [35], amphiphilic polyion complexes [36], organic-inorganic hybrids [37], ligand-stabilized metal nanoparticles (NPs) [38,39], and surfactant-encapsulated polyoxometalates [40]. They not only studied the formation mechanisms of the films, but also applied these films to various applications, such as separation membranes [41], superhydrophobic materials [42], photonic or optoelectronic devices [43], cell-culturing substrates [44,45], and micropatterning templates [46,47,48].

**Figure 2:**
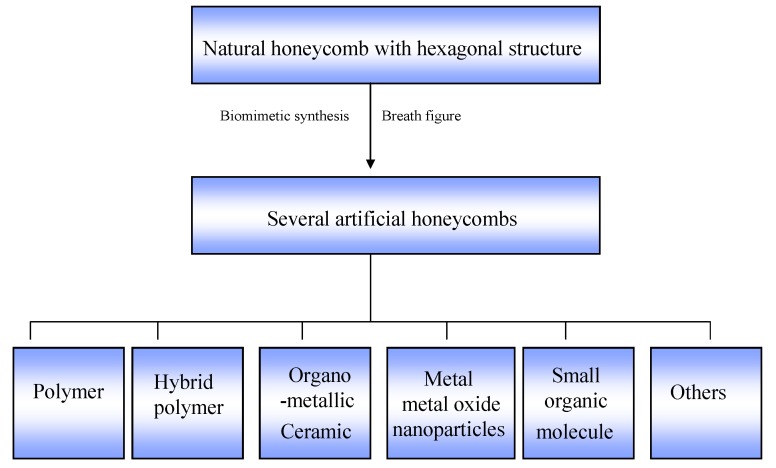
The design and fabrication of several artificial honeycombs whose inspiration comes from the natural honeycomb.

As mentioned above, natural honeycomb has a hexagonal structure which can provide inspiration for preparing two-dimensional (2D) hexagonal patterns. Currently, the 2D hexagonal structure fabricated by the BF method is similar to the natural honeycomb structure. So we named these 2D hexagonal patterns as bio-inspired honeycomb structures. The aim of the present review is to summarize the advances in the fabrication materials of honeycomb structure films, prepared by the BF method. We have chosen to discuss the literature that focuses on the following aspects (See Figure 2): honeycomb structured polymer films obtained by the BF method; hybrid honeycomb polymer films prepared by either self-assembly of hybrid NPs or growth of inorganic materials from precursors of NPs or directly from the honeycomb film; formation of organometallic and ceramic bubble arrays; formation of metal/metal oxide NPs honeycomb structure films; formation of small organic molecular honeycomb patterned films; others including honeycomb patterned films from DNA, graphene and even living bacteria.

## 2. Formation of Honeycomb Patterned Polymer Films

In 1994, Francois *et al*. used star and rod-coil polymers to obtain the honeycomb film by the BF method. At the beginning, the work in this field mainly focused on changing polymers, solvents and substrates to achieve all kinds of honeycomb structure films. Here the used polymer materials will be classified in detail.

### 2.1. Starlike and Graft Polymers

Star polymers are constitutive of several polymer chains attached at one end to only one branching point serving as the core. A series of starlike and grafted polystyrenes (PS) were investigated by Stenzel *et al*. [34,49,50,51,52,53]. When the CS_2_ solution containing the polymer was cast on the substrate under moist air, all of these polymers formed honeycomb structures. They investigated the influence of polymer concentration, molecular weight, and the number of arms on the pore size of the honeycomb structure. The results demonstrate that the more arms attached to a specific core, then the smaller the pores will be. Pore size decreases with increase in concentration. The glucose- and carboxy-terminated PS can easily form honeycomb structures of excellent quality, while ester-group-terminated PS cannot, when cast from CS_2_. When the blue-fluorescent star-poly(5-phenyl-8-(4-vinylphenyl) quinoline is utilized to fabricate honeycomb structures [53,54], breath-figure arrays (BFAs) of superior quality can be obtained. The pore sizes ranged from 150 nm to 1 μm when cast from CS_2_ under humid (85% H_2_O) conditions. The pore size also can be manipulated by the velocity of the air flow. Larger pores can be obtained from very low air velocities and confocal fluorescence pictures of their arrays have been observed. The fluorescence image of BFAs was patterned, with the maximum fluorescence intensity at 550 nm coming from the rims of the pores. This effect was interpreted as orientation of the polymer chains such that their fluorescent parts are closer to the surface of the pores. In addition, crosslinked star polymers are obtained by co-grafting styrene and divinylbenzene into a microgel by an arm-first approach [52]. Honeycomb structures can be formed by using these microgels successfully. Properties, such as order and monodispersity, are mainly dependent on the crosslinking time and the molecular weight.

### 2.2. Block Copolymers

**Figure 3:**
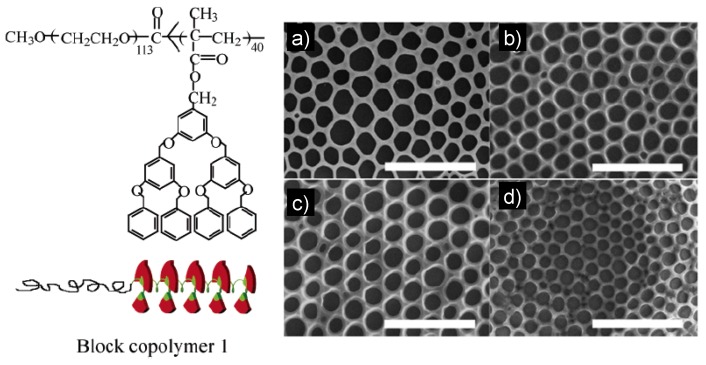
SEM images of the honeycomb structure of films prepared at different relative humidities (RHs): (**a**) 95%; (**b**) 90%; (**c**) 85%; (**d**) 80%. Other conditions: block copolymer 1 concentration, 0.75 mg/mL; spreading volume, 40 μL; temperature, 18 °C. The bar is 10 μm [60].

A block copolymer consists of two or more chemically different polymer segments or blocks connected by a covalent linkage. In recent years, several research teams [26,35,46,55,56,57,58,59] have reported such a regular hexagonal microporous structure film from rod-coil block copolymers, in which the rod blocks were mainly aromatic, conjugated rigid chains with small rod dimensions. It was indicated that polystyrene-co-poly(2,3,4,5,6-pentafluoro styrene) (PS-co-PPFS) copolymer porous films can be fabricated using CS_2_, CHCl_3_ and CH_2_Cl_2_ as solvent, respectively. However, compared to PS porous film, PS-co-PPFS copolymer porous films showed less ordered pore structures with two different average pore sizes. In addition, dendronized polymers are rodlike in shape, and the polymer backbone may form supramolecular helical structures containing a mixture of left- and right-handed helices in a condensed state due to the steric hindrance imposed by the bulky dendritic side groups attached to each repeating unit (Figure 3). Furthermore, dendronized polymers have larger rod dimensions and are semi-rigid. As far as we know, the honeycomb-like films are fabricated from such a rod-coil block copolymer containing a dendronized block. Several influencing factors on the formation of the different honeycomb structures, such as the concentration of the copolymer solution, the relative humidity in the atmosphere and the substrates, were investigated schematically (Figure 3). This work raised the possibility that such structure could be formed in block copolymers and extended the family of source materials. Recently, novel triblock copolymers with self-complementary hydrogen-bonding units were synthesized by using reversible addition fragmentation transfer polymerization. These polymers formed noncovalently crosslinked polymer particles and showed an aggregation behavior by intermolecular and intramolecular interactions. Well-ordered hexagonal microstructures were prepared by the BF technique with these triblock copolymers [60].

### 2.3. Amphiphilic Polymer

**Figure 4:**
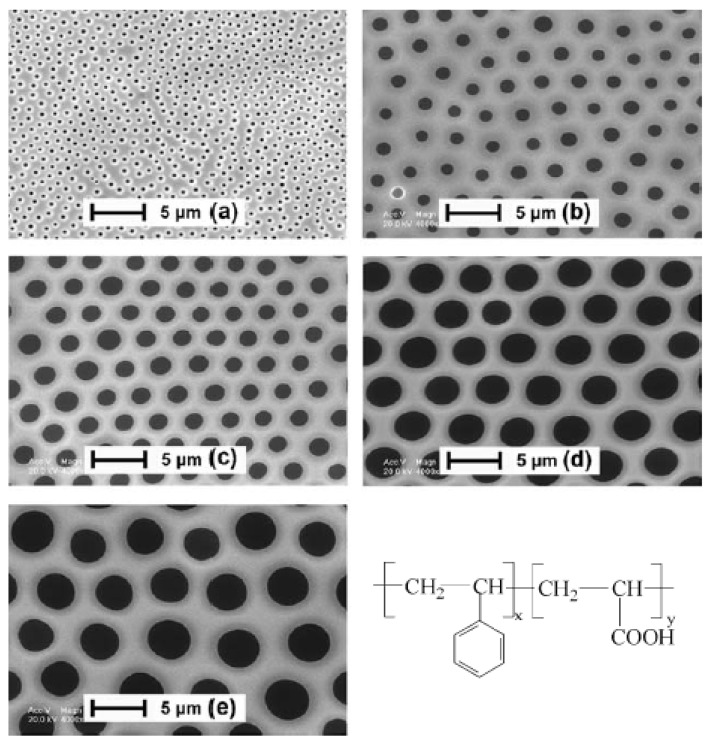
SEM images of the PS-b-PAA films prepared under different relative humidities. Solid substrate: glass slide; volume of PS-b-PAA/THF solution: 10 mL; solution concentration: 10 mg mL^−1^; relative humidity: (**a**) 60%; (**b**) 74%; (**c**) 80%; (**d**) 84%; (**e**) 94%. The structures of PS-b-PAA amphiphilic copolymer. x and y are the numbers of PS blocks and PAA blocks, respectively [32].

Amphiphilic polymers are composed of hydrophilic and hydrophobic parts. Formation of BFAs requires that the solutes can prevent water droplets from coalescing. Numerous studies have shown that this requirement can be met by using amphiphilic polymers [61,62]. Both amphiphilic polymers [32,63,64,65] and polymers with polar groups at the chain ends [25,26,66,67,68] tend to stabilize the condensing water droplets against coalescence (Figure 4). Shimomura *et al*. [47,69,70,71,72,73] demonstrated that amphiphilic polymers can be used as a second component or as additives to induce the formation of honeycomb structures with hydrophobic polymers. Fukuhira *et al*. [74,75] reported that phospholipids can be employed as biocompatible surfactants to fabricate biodegradable honeycomb patterned films, and they also proposed that interfacial tension between a water droplet and the polymer solution governs the formation of this patterned stucture. In addition, Nomura *et al*. [76] reported the fabrication of honeycomb-patterned thin films of PS and amphiphilic calixarene derivatives.

## 3. Formation of Hybrid Honeycomb Polymer Films

In nature, the most significant hybrid system examples can be found [77,78,79]. For instance, bio-mineralization is the natural hybrid process in which living organisms assemble to form solid nanostructures from existing inorganic and organic compounds. The resulting organism is described as ‘biohybrid’ in which biomolecules and inorganic components are intimately associated. The improved properties of the hybrid materials are not only a result of the simple combination of all the kinds of material intrinsic properties, but also the new dimensional arrangements of the different species. Inspired by the natural hybrid system, the concept of hybrid, which is using different chemical materials and assembling them to obtain a new one with enhanced properties, can be applied to many different fields. A simple mixture of organic and inorganic materials is not enough to obtain excellent properties, so they must be organized in a specific way. In the following sections, the fabrication of hybrid honeycomb structure polymer films through a simultaneous self-assembly of polymer and NPs, and the *in situ* formation of hybrid particles from precursor will be discussed [80].

### 3.1. Self-Assembly of Polymer/Nanoparticle System

**Figure 5:**
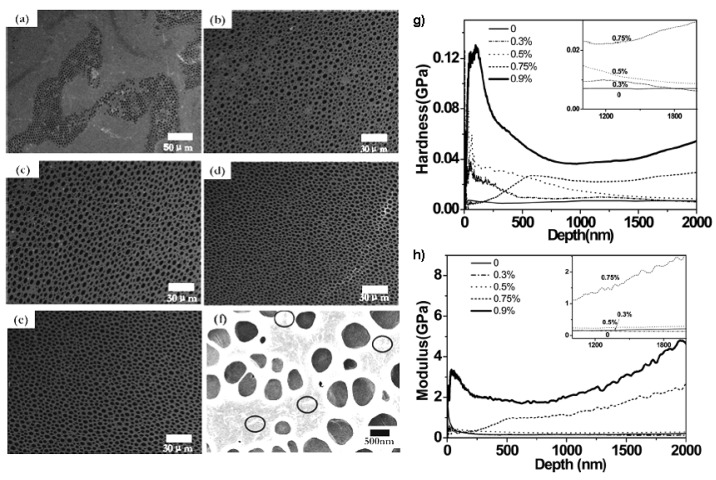
SEM images of the prepared honeycomb structure films. From image (**a**–**e**) the contents of nanoclay in the polymer solution are 0 wt%, 0.3 wt%, 0.5 wt%, 0.75 wt%, and 0.9 wt%, respectively; (**f**) High-magnification SEM image of; (**e**) showing the walls of the porous structure consisting of the clay layer with a thickness of ca. 50–80 nm and length of ca. 300–600 nm, marked by circles. From these figures, it can be seen that the pores have become more orderly and homogenous with the increase of nano-clay content (**g**) Hardness; and (**h**) modulus curves of polyimide-clay honeycomb structure films prepared with different clay content solution. Inset: Plot of the enlarged curves (with the nano-clay contents of 0 wt%, 0.3 wt%, 0.5 wt%, and 0.75 wt%) of (**g**) hardness; and (**h**) modulus with the range of 1000–2000 nm depth. From these figures, we know that the hardness and the Young’s modulus increased with increasing clay content [92].

The formation of honeycomb structure films by the BF method can be combined with the self-assembly of polymer/NPs mixtures at the polymer solution-water droplet interface. The complete evaporation of the solvent and water induces the polymer/NPs blends to assemble into the walls of an array of micron-sized spherical pores. Hult *et al*. [81] reported a honeycomb structural formation of the poly(9,9’-dihexyl-fluorene) (PDHF)/polystyrene-grafted silica NPs (Si-graft-PS) blend system using the BF method. Blends of Si-graft-PS NPs with 10–60 wt% PDHF were dissolved in CS_2_ and the solution was cast onto a glass substrate under humid conditions (66%–85% humidity). After the CS_2_ and water droplets completely evaporated, highly ordered close-packed micro-porous films were obtained. With this concept, it is also possible to force the localization of amphiphilic zeolite crystals at the interface inside the pores. Vohra *et al*. [82] reported that a solution of poly[(9,9-dioctylfluorenyl-2,7-diyl)-co-(1,4-benzo-(2,10,3)-thiadiazole)] (PF8BT) random copolymer containing a carboxylic acid-functionalized oxonine-loaded zeolite crystals (OxZLCOOH) was used to prepare hybrid honeycomb films successfully. Confocal fluorescence microscopy clearly showed two different emissions from the borders of the pores and the rest of the film, which illustrated that the amphiphilic crystals moved toward the water droplet interface to stabilize it. The results indicated that two levels of organization were obtained. One is a regular hexagonal array of micro-pores in a polymer film, the other is a selective positioning of the zeolite at the borders of the pores [82]. Since Boker *et al*. [83] reported that the pore surfaces could be decorated by cadmium selenide (CdSe) quantum dots (QDs), several other NPs such as Fe_2_O_3_ [84,85], Fe_3_O_4_ [85], gold [82,83,84,85,86,87,88] or silver [84,87] and CdSe/CdS QDs [85] have been used to create highly ordered hybrid honeycomb films. The introduction of nanoparticles, which showed interfacial activity is of benefit to the honeycomb film formation by stabilizing the water droplets during the BF process. Nevertheless, some developments have still to be designed to create highly structured superhydrophobic and conductive films. Ji *et al*. [89] presented an elegant way to tune the localization of silica NPs inside a highly structured honeycomb film. Both hydrophilic and hydrophobic SiO_2_ NPs were used to prepare honeycomb-structured hybrid films, which shows that a honeycomb patterned structure can be successfully formed regardless of particle wettability. The hydrophilic original NPs were basically adsorbed onto the pore surface while hydrophobic octadecyltrimethoxysilane-modified particles were assembled on the interior walls. Due to the hydrophilic nature of raw silica particles, they remained mainly located in the water phase during the early stage of the BF process. For the hydrophobic functionalized silica NPs, the precipitating polymer preferentially adsorbed around the droplet, hence resulting in localization of the hydrophobic NPs inside the walls. Moreover, organic electronic devices and photonic bandgap materials can be achieved by mixing organic conductive NPs such as carbon nanotubes (CNT) [87,90] or fullerene C60 [91] with conjugated polymers. Recently, our research team [92] also reported a kind of hybrid polymer ordered porous honeycomb structure film with enhanced mechanical strength and low density. The film was fabricated with polyimide as a basic structure and nano-clay as the enhanced layer in the honeycomb wall borders, and this mimics the multi-scale structure of natural honeycombs (Figure 5). After examining the mechanical properties of the honeycomb structures with different contents of clay, the results show that the hardness of the honeycomb films increased with increasing clay content, and reached a maximum value of 0.037 GPa, which was about five times that for the honeycomb film without clay. Because of the existence of the porous structure, the bulk density of the multiscale bio-inspired honeycomb structure films decreased and the porosity increased by 45.6%. Therefore, this kind of honeycomb structure, with high mechanical strength and low density, is considered to have wide applications in the areas of tissue engineering, aeronautical materials, separation films in lithium-ion batteries, and so on. Thus, the self-organized hybrid honeycomb films showed the combined properties of both the NPs as well as the ordered structures. Such hybrid films can be used as new photonic band gap materials [85], light-emitting devices [82] or magnetic patterned surfaces [84,85].

### 3.2. *In situ* Formation of the Hybrid Honeycomb Films

**Figure 6:**
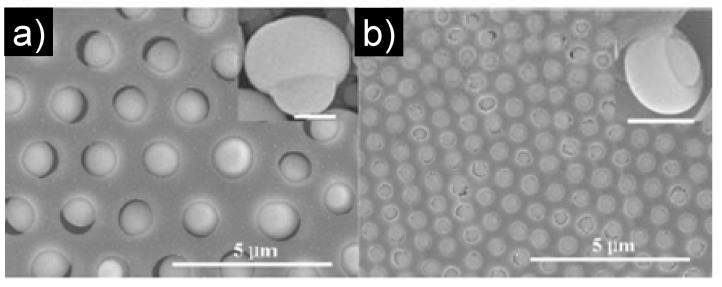
SEM images of the obtained composite film from the TiCl_4_/PS/CHCl_3_ solution with different concentrations of TiCl_4_: (**a**) 0.3% v/v; and (**b**) 0.4% v/v (PS, 1 wt %, relative humidity, 30%). Insets in (**a**) and (**b**) are the magnified mushroom-like particles, respectively. Scale bar = 0.5 μm [93].

Mixtures of polymer and metallic precursors of NPs have been used to create micro-patterned honeycomb structure hybrid polymer films by the BF method. The polymer helps the formation of the honeycomb film with micron-sized pores and the walls are filled with the precursors of NPs. Thus, highly structured hybrid honeycomb polymer films can be created *in situ*. Li *et al*. [93] fabricated patterned “bead in pore” composite film with hemispherical or mushroom-like TiO_2_ microparticles lying in the holes of a honeycomb-like polystyrene matrix (Figure 6) from the TiCl_4_/PS/CHCl_3_ solution via the BF method. The TiCl_4_ precursor was located inside the condensed water droplets which acted as “microreactors” for the TiCl_4_ hydrolysis. It is a very simple way to prepare hemispherical or mushroom-like TiO_2_ microparticles and to obtain the hexagonally nonclose-packed arrays of asymmetrical particles with or without a polymer matrix. This method is versatile in that other polymers also can be employed, and other hemispherical or mushroom-like particles may be obtained by using the corresponding precursors. It opens a new way to fabricate asymmetrical inorganic particles and their ordered arrays, which may find applications in photonic crystals, biomedicine, catalysis, and so on. The polymer-nanoparticle composite film can also be prepared by a one-pot reduction of metallic ions during the film formation as described by Jiang *et al*. [94]. In this work, by an *in situ* reduction method, the blended solution of silver NPs and polyurethane (PU) influenced the formation of regular pore arrays on the surface, which depends upon the humidity levels and the content of Ag NPs and polymer. The results showed that addition of Ag NPs promoted honeycomb structure formation under low humidity (<30% humidity). Chen *et al*. proposed an alternative strategy to fabricate functional honeycomb-patterned films with controllable pore sizes via BF based on ionomers [95], which are polymers with a small mole fraction of chemically bonded ionic moieties. In this case, well-defined poly(methyl methacrylate)/cadmium acrylate (PMMA/Cd(AA)_2_) ionomers were synthesized via radical polymerization and Cd(AA)_2_ (two acrylates bonded to one Cd^2+^) acted as a cross-linker. Subsequently, ordered porous films were successfully deposited on glass substrates from the ionomer solutions under a humid environment. The pore sizes of these films could be simply adjusted by changing the experimental parameters such as the concentrations of the ionomer solutions or the molar ratios of monomers. Moreover, depending on the chemically bonded Cd^2+^ ions in the polymer matrixes, *in situ* generation of CdS NPs was possible by exposing the chloroform solution of PMMA/Cd(AA)_2_ to a H_2_S atmosphere. Evaporation of the solvent yielded honeycomb-patterned PMMA/CdS QDs-polymer films which showed favorable fluorescence in the absence of quenching, characteristics of the good dispersion of the NPs in the polymer film. 

## 4. Formation of Small Organic Molecular Honeycomb Patterned Films

**Figure 7:**
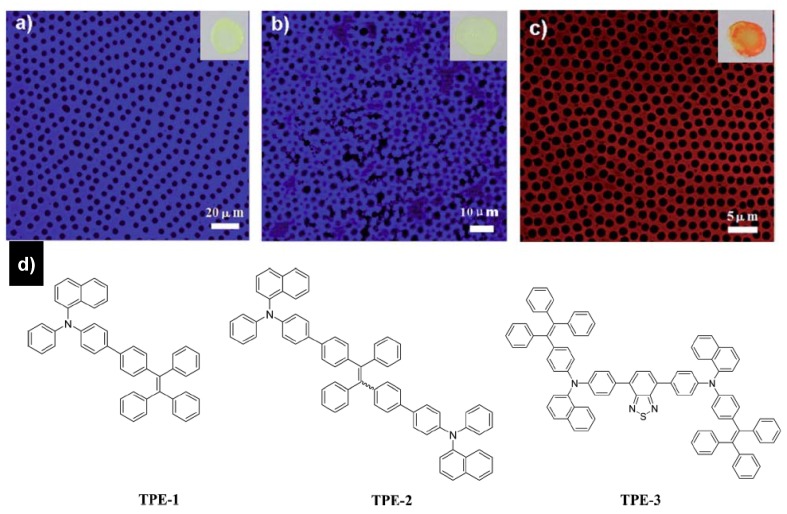
The fluorescent images of the as-prepared honeycomb structure films from (**a**) TPE-1; (**b**) TPE-2; and (**c**) TPE-3, The insets are the corresponding photographs of the films; excitation: 405 nm; (**d**) molecular structures of tetraphenylethene derivatives used in experiments [99].

Formation of macroporous honeycomb structures using polymers and hybrids have been well studied. However, there are few studies about the fabrication of honeycomb structures using small molecules as building blocks by the BF method. Kim *et al*. [96] first reported the application of the BF method to a small molecule. The photoresponsive organogelator self-assembled into supramolecular fibrillar networks and further a hierarchically ordered honeycomb structure. Babu *et al*. [97] also reported the formation of hierarchical macroporous structures from an amino acid linked p-conjugated organogelator. Recently, a new organogelator was synthesized and large-scale ordered honeycomb patterns were also observed [98]. Furthermore, we reported the successful fabrication of honeycomb structure by the BF process from derivatives of the small molecule tetraphenylethene (TPE) (Figure 7), showing an extraordinary phenomenon of aggregation-induced emission (AIE) [99]. In this process, TPE derivatives with the twist and non-planar substituted groups are chosen. TPE units become amorphous more easily than crystalline, which is critical for gaining viscosity and stabilizing the water droplets during evaporation. The fluorescence data including micrographs and spectra indicate that these honeycomb structures are highly emissive due to the AIE feature of TPE derivatives. These structures lead to a small red-shift in photoluminescence compared to the smooth film. The success of fabricating the honeycomb structure of TPE derivatives may, for certain applications, represent an advance with respect to the more commonly used polymers, due to the inherent drawbacks of polymers such as phase separation and nonreproducibility of molecular weight distribution from batch to batch. These findings open up a new way for the development of honeycomb structure materials with small organic molecules. 

## 5. Formation of Organometallic and Ceramic Honeycomb Structures

**Figure 8:**
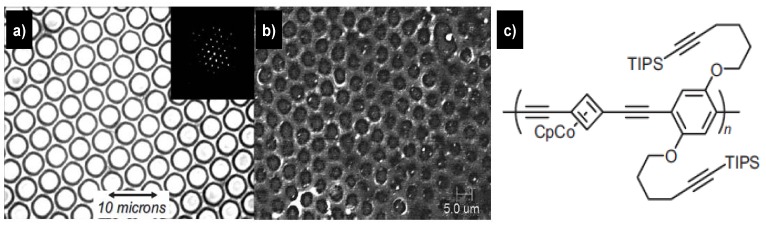
Bubble arrays from polymer PPE (**a**) before; and (**b**) after pyrolysis at 500 °C under nitrogen. The inset in (**a**) shows the hexagonal diffraction pattern of the array [100].

The structure of organometallic polymers can be optimized by employing different monomers and through polymer blending, which give them unique solubility and fusibility over classical ceramic materials. Thus organometallic polymers are suitable candidates as precursors for ceramics, such as SiC, Si_3_N_4_, AIN, BN or TiN. Pyrolysis can transform these polymers into the ceramic state. When the organometallic PPE (Figure 8c) was cast from CS_2_, a well-shaped honeycomb structure film formed (Figure 8) [100,101,102]. Due to the high content of silicon, carbon, and cobalt, BFAs of PPE do not melt, but form a highly crosslinked and insoluble ceramic material upon heating to 600 °C. In this case, the formed material is not just organic but ceramic in nature owing to the higher pyrolysis temperature. The ceramic yields are high: 88% for pyrolysis under nitrogen and 97% for pyrolysis under air. Electron-dispersive X-ray scattering gives the elemental composition of these ceramics. When the pyrolysis was performed under nitrogen a C, Si, Co ceramic formed, while during pyrolysis under air a Si, Co, O ceramic was obtained in which all of the carbon was burned out. The originally interconnected holes in the honeycomb structure collapsed into ceramic wells. Heating a thermoset material should enable fabrication of other permanent structures. When a honeycomb structure of carboxylated nitrocellulose, cast from amyl acetate, was treated with Ni^II^ acetate or Co^II^ acetate in water, clean ion exchange took place without disruption of the array. Pyrolysis of the cobalt- or nickel-containing bubble layer at 700 °C gave flat honeycomb structures of 60 nm in height, indicating a 25% ceramic yield. The metallized carbon networks show interesting conducting properties [103]. Ma *et al*. [104] also reported a similar facile methodology to prepare highly ordered ceramic micropatterns on solid substrates by pyrolyzing UV cross-linked polymer microporous films formed by a polydimethylsiloxane-b-polystyrene block copolymer and tetrabutyl titanate Ti(OC_4_H_9_)_4_, as a functional precursor of TiO_2_.

## 6. Formation of Metal and Metal Oxide Nanoparticle Honeycomb Structure Films

### 6.1. Formation of Metal Nanoparticle Honeycomb Structure Films

**Figure 9:**
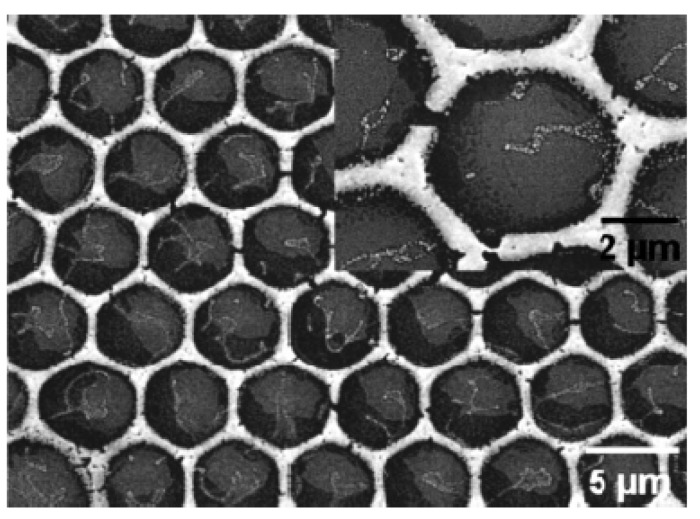
SEM image of a 5.36 g/L Au NPs deposited sample annealed at 400 °C for 60 min. The inset shows a magnified view of one of the hexagons [106].

Dodecanethiol-coated gold NPs cast from toluene in moist air can form regular honeycomb structures. When the casting was performed at high air velocities with a horizontally tilted nozzle, the formed bubble arrays were not spherical and hexagonally ordered but were elliptically distorted, and showed more of a brick-wall-type rectangular arrangement [105]. Korgel *et al*. [38,106] have investigated the BF formation of dodecanethiol or perfluoro-thiol stabilized gold NPs (5 nm) cast from Freon-type solvents or CS_2_. Casting of trioctylphosphine-stabilized InMnAs NPs from chloroform was explored as well. Hexagonal bubble arrays with bubble dimensions from 350 nm to 5 μm formed upon evaporation in a humid environment. Korgel investigated the mechanism of bubble formation in these systems and found that the gold nanocrystals do not reduce the interfacial tension between Freon and water [38]. However, poorly solvated and heavily washed gold NPs reduce the water/nanoparticle contact angle from 94° to 83° and modulate the Freon/water contact angle. In this case, the Freon/water contact angle decreased from >150° to 112°, suggesting that the NPs stabilize a water-in-Freon “Pickering” emulsion [107]. The decreased contact angle leads directly to the observed large-area, hexagonally ordered preparations of the droplet rafts. When such gold hexagonal arrays were pyrolyzed at 400 °C, the organic ligand was burned off and gold-honeycomb arrays were obtained (Figure 9) [106]. Besides the gold NPs, heptacosafluoro-pentadecane-1-thiol stabilized silver NPs (2 nm) can also form highly ordered bubble arrays whose diameter is 2–3 μm [108]. The polymer’s concentration becomes sufficiently high due to evaporation of the solvent. This arrangement is fixed and the immobilized NPs stay at the interface. The phenomenon of interfacial segregation of NPs by immiscible liquids had been described earlier and was used as a guiding principle to construct these hierarchically self-assembled BFAs [107,109].

### 6.2. Formation of Metal Oxide Nanoparticles Honeycomb Structure Film

**Figure 10:**
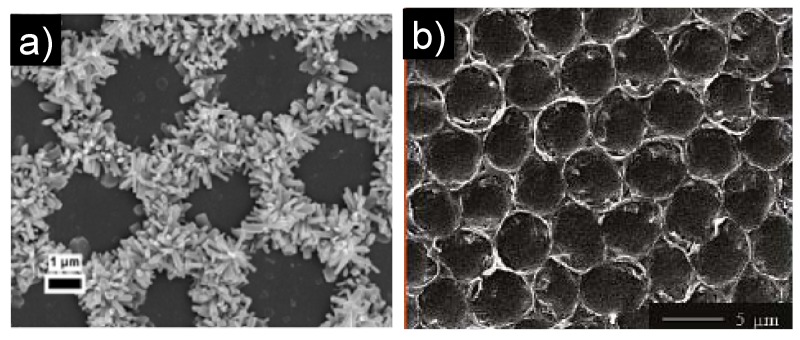
(**a**) SEM image of hydrothermal ZnO nanorod arrays grown from Zn(acct)_2_ honeycomb structured pattern.[48]; (**b**) SEM image of the PS/TTIP as TiO_2_ precursor honeycomb structured film after 24 h UV light treatment and then calcined at 550 °C [111].

As mentioned above, Karthaus *et al*. reported that a patterned zinc oxide (ZnO) containing polymer honeycomb film was achieved using ZnO NPs precursor and a poly(styrene-co-maleic anhydride) (PS-co-PMAh) by the BF method [110]. They also described the method to create a pure ZnO honeycomb film with photocatalytic properties. An amphiphilic polyion complex (PIC) was first prepared by mixing equimolar amounts of an aqueous solution of poly(styrene sodium sulfonate) (PSSNa) and a vesicular emulsion of bishexadecyldimethyl ammonium bromide. The PIC was further mixed with zinc complex, acetylacetonate hexahydrate Zn(acac)_2_, in various ratios. The 5:1 mixing ratio of Zn(acac)_2_:PIC produced stable honeycomb structures after pyrolysis. The pyrolysis obviously led to shrinkage of the film, but the integrity of the in-plane structure of the honeycomb film was well preserved. The organic material in the rim decomposed, and thus the rim got thinner but the pore-pore distance remained similar. Zhao *et al*. used the same process to introduce a honeycomb structured hybrid film into inorganic photoactive TiO_2_ film [111]. In their study, a solution of PS with titanium tetraisopropoxide (TTIP) as TiO_2_ precursor was prepared. They demonstrated a simple and effective vapor phase hydrothermal modification method (calcination at 550 °C for 2 h) which is capable of transforming a honeycomb structured hybrid film into a photoactive TiO_2_ film without dismantling the originally templated three-dimensional structure (Figure 10). The preservation of the organic/inorganic hybrid film structure during its conversion to pure inorganic film by means of pyrolysis was ensured further [111].

Li *et al*. [48,112] proposed a very interesting way to create a hierarchical structured honeycomb hybrid film. A classical procedure was used to prepare a honeycomb structure film from an amphiphilic diblock copolymer, polystyrene-b-poly(acrylic acid) (PS-b-PAA) solution in CS_2_, containing ferrocene or zinc acetyl acetaonate (Zn(acct)_2_) as chemical precursors of ZnO NPs. This film’s formation was followed by a photochemical cross-linking of the copolymer under UV light. After 4 h of UV exposure, the cross-linked film was heated to 450 °C within 2 h and held for another 5 h under air atmosphere. During the pyrolysis, the functional precursor turned into oxide and replaced the polymer skeleton, leading to functional inorganic patterns (ZnO). Indeed, such functional inorganic honeycomb films were used to grow ZnO nanorod arrays from ferrocene (using acetylene flow at 750 °C) and zinc precursors, respectively (Figure 10) [48]. In addition, Wu *et al*. [40,113,114,115,116,117,118] have shown that surfactant-encapsulated polyoxometalate (POM) clusters are a new type of building block for fabrication of honeycomb structures. A series of hydrophobic surfactant-encapsulated clusters prepared from POM with different compositions, shapes, and sizes, are able to self-assemble into ordered honeycomb structures [113,117]. Sakatani *et al*. [119] reported the fabrication of macroporous films using surfactant-modified NPs (SiO_2_, TiO_2_, Co, and CdS) based on the BF method. Hierarchically porous materials made of metallic oxides can also be obtained through calcinations of the macroporous films. Xu *et al*. [120] reported that hierarchically ordered two-dimensional architectures can be prepared from various nanocrystal building blocks. 

## 7. Other films including BFAs from DNA, Graphene and even Living Bacteria

**Figure 11:**
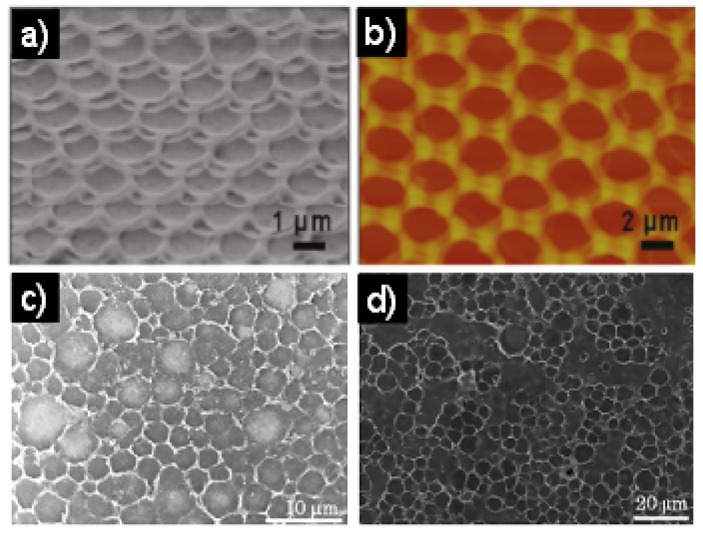
Honeycomb-patterned film prepared from 0.75 mg/mL of DNA-DTDA complex: (**a**) SEM image; (**b**) AFM image [121]. Typical SEM images of the honeycomb GO-complex films fabricated from the toluene solution (1.0 mg/mL) on glass substrates under (**c**) 80%; and (**d**) 90% RH [122].

Besides the above-mentioned, some special material honeycomb structure films were also reported. Wu *et al*. [121] reported the self organization of DNA into honeycomb structures on solid substrates by a simple solution casting of DNA-ditetradecyldimethylammonium (DTDA) complex at high relative humidity (Figure 11a,b). This work investigated the effects of the substrate type, DNA-DTDA complex concentration, solvent type, and two different DNA-surfactant complexes on the morphologies of the microporous films. Furthermore, the dye molecule rhodamine B (RhB) was loaded into the DNA-DTDA complex to obtain a fluorescent honeycomb film. The present research gives a convenient route to fabricate DNA-based honeycomb films, establishes the multicomponent self-assembly in honeycomb films to endow the DNA-surfactant complex with fluorescent properties, and provides the films with increased functionality. Nakashima *et al*. [122] prepared the graphene honeycomb films self-assembled on glass substrates from the graphene oxide (GO) specified organic solution. The soluble GO materials in many organic solvents including low dielectrically nonpolar organic solvents such as toluene and hexane can be easily obtained with the aid of tridodecylmethylammonium chloride (TDMAC), and this would greatly extend the fundamental research and novel applications of graphene materials. Under high relative humidity conditions, the cast films from the GO complex in toluene on the glass substrates have honeycomb super-structures with ordered macropores (Figure 11c–d), which are very useful in many areas of nanoscience and technology, including nanoelectronics, nanodevices, catalysts, and sensors, *etc*. In addition, Shimomura attempted to fabricate honeycomb-patterned bacterial cellulose (BC) by controlling the bacterial movement using an agarose film scaffold with honeycomb-patterned grooves (concave type) [123]. The patterned agarose film was prepared by three steps. The first was transcription of a honeycomb-patterned polycaprolactone film template with polydimethyl siloxane. When the bacteria were cultured on the scaffold under atmospheric conditions, only bacterial proliferation was observed. Honeycomb-patterned BC was obtained when cultured under a humid CO_2_ atmosphere. Electron diffraction and polarized microscopic observation showed that the patterned BC is comprised of the well defined cellulose. In another attempt to fabricate honeycomb-patterned BC, the bacteria were cultured on the patterned cellulose and agarose film with convex type of honeycomb. This culture yielded no honeycomb-patterned BC. Therefore, concave type honeycomb scaffold is more suitable to fabricate honeycomb-patterned BC.

## 8. Conclusions

With the emergence of the BF method, work mainly focused on the fabrication of honeycomb structure films from different kinds of polymers, different solvents and different substrates. Then with the development of the BF method, the fabrication material category extended from polymer to hybrid materials, organometallic/ceramic, metal/metel oxide nanoparticles, small organic molecules, DNA, graphene and living bacteria. The resultant honeycomb structures have been widely used in many fields, such as separation membranes, superhydrophobic materials, photonic or optoelectronic devices, cell-culturing substrates, and micropatterning templates. However, there are several outstanding issues such as reliably producing very small or very large pores, constructing from any polymeric material, preparing a large area of uniform honeycomb structure and positioning the three-dimensional honeycomb material growth. Effective backfilling to obtain compounded micro- and nano-scale materials presents exciting challenges to be met in the future.
